# Adult Rome IV Disorders of Gut–Brain Interaction in a Pediatric Population †

**DOI:** 10.3390/children13030438

**Published:** 2026-03-23

**Authors:** Natali González Rozo, Carlos Alberto Velasco-Benítez, Michelle Higuera Carrillo, Daniela Alejandra Velasco-Suárez

**Affiliations:** 1 Faculty of Medicine, Department of Pediatrics, Universidad de Pamplona, Cucuta 540001, Colombia; natali.gonzalez@unipamplona.edu.co; 2 Hospital Universitario Erasmo Meoz, Cucuta 540001, Colombia; 3 Faculty of Medicine, Department of Pediatrics, Universidad del Valle, Cali 760001, Colombia; carlos.velasco@correounivalle.edu.co; 4 Faculty of Social Sciences Doctorate in Educational Innovation, Universidad de Santander, Bucaramanga 680001, Colombia; 5 Faculty of Medicine, Department of Pediatrics, Universidad Nacional de Colombia, Bogota 110111, Colombia; mihiguerac@unal.edu.co; 6 Faculty of Medicine, Department of Pediatrics, Universidad El Bosque, Bogota 110111, Colombia; 7 Gastrohnup Research Group of the Universidad del Valle, Cali 760001, Colombia

**Keywords:** disorders of gut–brain interaction, Rome criteria, proctalgia fugax, quality of life

## Abstract

**Background**: Disorders of the gut–brain interaction (DGBIs) constitute a group of functional conditions widely described in adults; however, some of these have not been included in pediatric Rome criteria, despite the fact that they may manifest during childhood. Early identification of these conditions is relevant due to their clinical/psychosocial impact as well as their effect on quality of life. The aim was to determine the prevalence and associated factors of some DGBIs described in adults according to the Rome IV criteria in pediatric population. **Methods**: An observational/prospective/cross-sectional study was conducted in toddlers, school-aged children, and adolescents from three Colombian cities. The adapted Questionnaire for Pediatric Gastrointestinal Symptoms Rome IV (QPGS-IV) using adult criteria was applied, along with quality-of-life scales and PROMIS for anxiety/depression. Descriptive uni/bivariate analyses were performed as well as a multivariate logistic regression model. **Results**: A total of 704 participants were included (13.7 ± 2.8 years old). The prevalence of DGBIs described in adults according to QPGS-IV was 5.8%, with proctalgia fugax being the most frequent. In the bivariate analysis, race, school/social absenteeism, depressive traits, and impaired quality of life were significantly associated. In the multivariate model depressive traits (OR = 4.08; 95%CI = 1.82–9.12; *p* = 0.001), school (OR = 2.51; 95%CI = 1.06–5.98; *p* = 0.036), and social absenteeism (OR = 4.04; 95%CI = 1.70–9.62; *p* = 0.002) were the factors independently associated. **Conclusions**: These adult DGBIs, according to the QPGS-IV, can occur in pediatric populations and are closely related to psycho-emotional and functional factors. These are mainly associated with depression and school/social absenteeism, supporting the need for a biopsychosocial approach and a revision of the pediatric diagnostic criteria.

## 1. Introduction

Disorders of the gut–brain interaction (DGBIs) constitute a group of conditions arising from the interplay of altered gastrointestinal motility and visceral sensitivity, dysfunction of the immune system and intestinal mucosa, imbalances in the gut microbiota, and changes in the perception and central processing of gastrointestinal signals by the central nervous system [1], without identifiable structural or biochemical abnormalities that explain these symptoms [2].

In childhood, DGBIs are associated with a considerable symptom burden, accompanied by psychological distress, reduced quality of life, school and social absenteeism, increased healthcare costs, and parental work absenteeism [3], moreover, they are associated with a higher likelihood of persistence or progression of the disorder into adulthood [4]. It is estimated that approximately 25.0% of children with recurrent abdominal pain subsequently develop irritable bowel syndrome [3].

The Rome Committee periodically emits criteria that facilitate the diagnosis and identification of the pediatric population with DGBIs [5]. Over the past three decades, the Rome criteria have been the main diagnostic tool for these disorders in children, being periodically updated based on recent scientific evidence and expert consensus; the most recent version, Rome IV, was published in 2016 [1] and we are currently on the verge of the publication of the next version, scheduled for May 2026.

Some DGBIs are well-defined in the adult population but have not yet been incorporated into the pediatric Rome criteria. Such is the case of functional dysphagia [6,7], functional diarrhea [2,8], functional chest pain [9], functional biliary pain [10] as well as functional heartburn and proctalgia fugax [11].

According to adult criteria, functional dysphagia is characterized by the sensation of abnormal passage of the food bolus through the esophagus in the absence of structural, mucosal, or motor abnormalities [6,7,12]. Functional diarrhea is characterized by the recurrent presence of liquid or semi-liquid stools without predominant abdominal pain or evidence of structural abnormalities [8]. It is a common disorder in young children, with studies conducted in different countries reporting a prevalence ranging from 1.9 to 2.5% in children under one year of age [2]. Functional chest pain presents as recurrent, unexplained retrosternal pain of presumed esophageal origin, not explained by mucosal or motor disorders, and distinct from heartburn-related pain, occurring at least once per week [9], while functional biliary pain is characterized by constant pain located in the epigastrium or the right upper quadrant of the abdomen, lasting at least 30 min, similar to biliary colic, in the absence of gallstones or biliary abnormalities [10], and functional heartburn is defined as an unpleasant sensation or retrosternal burning pain that does not respond to optimal antisecretory treatment, in the absence of gastroesophageal reflux disease, histopathological alterations of the mucosa, relevant motor disorders, or other structural causes that explain it [6]. Finally, proctalgia fugax is characterized as chronic or recurrent rectal pain, described as pain or discomfort lasting at least 30 min, with no evidence of a structural or systemic cause [11]. According to the Rome IV criteria for adults, the diagnosis of these disorders requires that symptoms have been present for at least three months, with symptom onset at least six months before evaluation, and that there be no evidence of a structural or metabolic disease to explain the symptoms [13]. The absence of pediatric diagnostic criteria for these conditions raises important questions about whether these disorders may already manifest during childhood but remain underrecognized due to the lack of age-adapted definitions. Emerging evidence suggests that the clinical expression of DGBIs may follow a continuum across the life course, with early manifestations occurring during childhood and adolescence before evolving into more clearly defined adult phenotypes. However, epidemiological data evaluating the presence of these adult-defined disorders in pediatric populations remain scarce, particularly in Latin America.

Therefore, the aim of this study was to determine the prevalence of selected adult DGBIs according to the Rome IV criteria using the Questionnaire for Pediatric Gastrointestinal Symptom Rome IV (QPGS-IV), adapted from adult criteria, in toddlers, school-aged children, and adolescents from public educational institutions in three Colombian cities.

## 2. Materials and Methods

A prospective, observational, cross-sectional study was conducted in toddlers, school-aged children, and adolescents aged 4 to 18 years old, from public educational institutions in three Colombian cities: Cúcuta (Andean Region), Maicao (Caribbean Region), and Corozal (Caribbean Region). After obtaining informed consent/assent from children older than 7 years old, data collection was initiated through the administration of the QPGS-IV questionnaire, adapted with additional items corresponding to the diagnostic model used in adults. The mode of administration varied according to the participant’s age: in children aged 4 to 9 years, it was completed by parents or caregivers whereas in children aged 10 years and older, the questionnaire was self-administered, in order to ensure appropriate comprehension of the questions and accurate reporting of symptoms. This adaptation allowed for a more in-depth exploration of the presence of symptoms and conditions that, although more frequently described in the adult population, may also manifest during childhood (functional dysphagia, functional diarrhea, functional chest pain, functional biliary pain, functional heartburn and proctalgia fugax) (Table 1). Consistent with the structure of the QPGS-IV and Rome IV pediatric diagnostic criteria, a minimum symptom duration of 2 months was used for symptom assessment. Although adult Rome IV criteria typically require a symptom duration of 3–6 months prior to diagnosis, the 2-month timeframe was maintained to ensure methodological consistency with the pediatric instrument applied in this study.

Although this adapted version has not undergone a formal psychometric validation process, its internal consistency was evaluated within the study population using Cronbach’s alpha. The overall questionnaire showed high internal consistency (α = 0.7620). When analyzed by domains, the following Cronbach’s alpha values were obtained: functional dysphagia (section I) α = 0.7568, functional diarrhea (derived from section C) showed moderate internal consistency (α = 0.4994), functional chest pain (section G) α = 0.6404, functional biliary pain (section F) α = 0.7393, functional heartburn (section H) α = 0.6246, and proctalgia fugax (section K) α = 0.8061.

Likewise, sociodemographic variables (age, sex, race and place of origin), clinical variables (history of COVID-19 and COVID-19 vaccination status) were collected.

Aspects related to school and social absenteeism were also considered, including the reasons that led children or adolescents to miss their usual activities, as well as the assessment of their quality of life using the Pediatric Quality of Life Inventory™ Questionnaire (PedsQL 4.0), which allows for the measurement of the impact of health problems across different dimensions of a child’s well-being. To explore psycho-emotional aspects, Patient-Reported Outcomes Measurement Information System (PROMIS) anxiety and depression were also applied to assess the presence of anxiety and depression traits in the studied population.

The statistical analysis included the calculation of measures of central tendency to describe the characteristics of the sample, as well as univariate and bivariate analyses to identify associations between variables. A multiple logistic regression analysis was also performed to determine the independent factors associated with the presence of adult DGBIs according to the Rome IV criteria. Results were expressed as odds ratios (ORs) with their corresponding 95% confidence intervals (95%CIs), with a *p* value < 0.05 considered as statistically significant.

This study was approved by the Ethics Committee of the Hospital Universitario Erasmo Meoz from Cúcuta, Colombia (approval No. 51-2024, dated 30 July 2024). In addition, written authorization was obtained from the institutional authorities of the participating educational establishments.

## 3. Results

**Figure 1:**
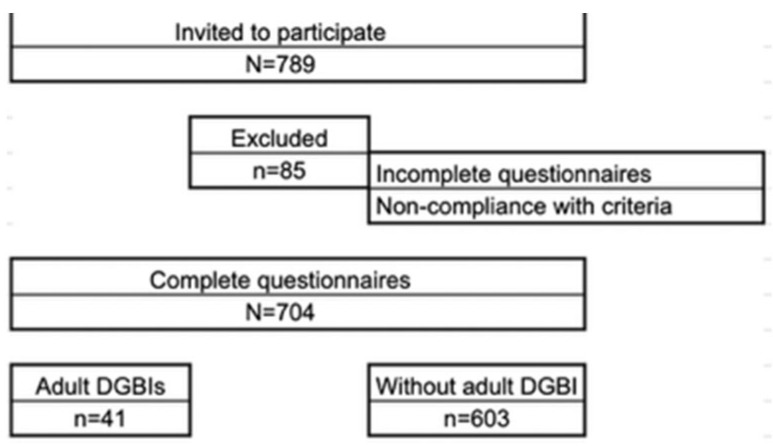
Study flowchart.

A total of 789 students from public educational institutions in the cities of Cúcuta (Andean Region), Maicao (Caribbean Region), and Corozal (Caribbean Region) were invited to participate. Eighty five were excluded due to incomplete questionnaires or failure to meet the inclusion criteria, resulting in 704 students included in the final analysis. Among the included participants, 41 students met the criteria for at least one adult DGBI (Figure 1).

### 3.1. DGBIs

According to the Rome IV criteria, 23.4% of students presented at least one DGBI (95%CI = 0.20–0.26), with functional constipation being the most frequent (18.0%; 95%CI = 0.15–0.21), followed by postprandial distress-type functional dyspepsia (2.4%; 95%CI = 0.01–0.03) and functional vomiting (1.0%; 95%CI = 0.00–0.02). A total of 2.7% of students presented more than one DGBI (95%CI = 0.01–0.04); among the most frequent overlaps were functional dyspepsia + constipation (1.1%; 95%CI = 0.00–0.02), followed by constipation + cyclic vomiting syndrome (0.6; 95%CI = 0.00–0.01). A total of 5.8% (n = 41) presented some DGBIs described in adults according to the Rome IV criteria (95%CI = 0.04–0.07): proctalgia fugax 2.8% (95%CI = 0.01–0.04) (n = 20), functional heartburn 1.0% (95%CI = 0.00–0.02) (n = 7), functional diarrhea 0.9% (95%CI = 0.00–0.01) (n = 6), functional dysphagia 0.6% (95%CI = 0.00–0.01) (n = 4), functional chest pain 0.4% (95%CI = 0.00–0.01) (n = 3), and functional biliary pain in 0.1% (95%CI = 0.00–0.00) (n = 1) (Table 2).

The main characteristics of all participants between 4 and 18 years old and the 41 children with adult-onset DGBIs according to the Rome IV criteria included in the study are presented in Table 3.

### 3.2. Possible Associations with the Presence of Adult DGBIs According to the Rome IV Criteria

The possible risk factors for presenting at least one adult DGBIs according to the Rome IV criteria included indigenous race (OR = 2.36; 95%CI = 1.02–5.06; *p* = 0.0170), school absenteeism (OR = 3.79; 95%CI = 1.90–7.60; *p* < 0.001), social absenteeism (OR = 5.90; 95%CI = 2.93–12.03; *p* < 0.001), depression traits (OR = 3.97; 95%CI = 1.73–9.49; *p* = 0.0002), and an altered quality of life (OR = 2.36; 95%CI = 1.09–5.04; *p* = 0.0135) (Table 4).

### 3.3. Logistic Regression Analysis

The multivariate logistic regression model, which included all variables that showed statistical significance in the bivariate analysis, showed that depressive traits and school and social absenteeism were the most important variables associated with the presence of adult DGBIs in the pediatric population, behaving as factors significantly related to this outcome (OR = 4.08; 95%CI = 1.82–9.12; *p* = 0.001, OR = 2.51; 95%CI = 1.06–5.98; *p* = 0.036 and OR = 4.04; 95%CI = 1.70–9.62; *p* = 0.002, respectively) (Table 5).

## 4. Discussion

In our study, the prevalence of DGBI was 23.4%, a result consistent with that reported in a recent meta-analysis [14]. Consistent with the current literature, the most frequently identified disorder was functional constipation, recognized as the most prevalent DGBI in the pediatric population [15]. Regarding the overlap of DGBIs, our study identified a prevalence of 2.7%, which is lower than that reported in previous studies conducted in Latin American pediatric populations. In particular, one study [16] reported an overlap prevalence of 8.4% using the Rome III criteria. This difference could be explained, at least in part, by the use of different diagnostic criteria, as the Rome IV criteria used in our study are more restrictive and have been shown to identify a smaller proportion of cases compared with Rome III. Additionally, methodological variations—such as the characteristics of the questionnaire used, the operational definition of overlap, the age range of the studied population, and the sociocultural context—may have influenced the lower frequency of overlap observed.

The results of this study provide evidence that several DGBIs traditionally considered exclusive to the adult population according to the Rome IV criteria can be identified in the pediatric age group, although with a relatively low prevalence (5.8%). This finding raises important questions regarding the current age-based delimitation of diagnostic criteria for DGBIs and suggests that a strict separation between pediatric and adult phenotypes may not fully reflect the physiopathological continuity of these disorders across the life course [17,18]. In contrast, population-based studies in adults have reported that up to 40.3% of the population presents at least one DGBI when the Rome IV criteria are applied, reinforcing the hypothesis that these conditions may manifest early with subclinical or incomplete expressions during childhood and later consolidate or become more evident in subsequent stages of life [19].

The higher frequency observed in adolescents supports the hypothesis that the clinical expression of DGBIs evolves progressively with age, possibly influenced by neurobiological, hormonal, and psychosocial changes characteristic of this stage [17,20]. These age-related differences may also reflect developmental changes in the gut–brain axis, including maturation of central pain processing pathways, hormonal modulation during puberty, and alterations in gut microbiota composition, all of which may influence visceral sensitivity and gastrointestinal motility. Previous studies have described that functional gastrointestinal symptoms in childhood may persist or evolve into more complex clinical presentations in adulthood, reinforcing the notion of a continuous spectrum rather than discrete entities [21,22,23]. Nevertheless, the lack of validated pediatric criteria for these disorders limits a direct comparison with other studies and underscores the need for longitudinal research. The finding of proctalgia fugax as the most frequent adult DGBI in our pediatric cohort is noteworthy, as this functional anorectal disorder is usually underrepresented in pediatric epidemiological studies, likely due to difficulties in its identification in routine clinical practice and the underreporting of anorectal symptoms [24,25]. In the adult population, proctalgia fugax has been reported with a variable prevalence, ranging from approximately 8.0 to 18.0% in population-based studies, suggesting that it is a relatively common but underdiagnosed condition [26]. This discrepancy between the frequency reported in adults and its limited description in pediatrics reinforces the hypothesis that the use of structured questionnaires based on the Rome IV criteria may have contributed to a greater detection of these symptoms, highlighting the importance of standardized instruments for a more comprehensive characterization of the spectrum of DGBIs in the pediatric age group [27].

In our study, the prevalence of functional diarrhea was 0.9%, a figure lower than that reported among school-aged children and adolescents in other population-based studies, where a prevalence near 1.7% has been described [18]. This frequency contrasts more markedly with what has been observed in the adult population, in which functional diarrhea reaches significantly higher prevalences, estimated between 3.6% and 5.3% according to population-based studies using the Rome IV Criteria [28]. These differences suggest that the clinical expression of functional diarrhea may increase with age, either due to true progression of the disorder across development or to a greater ability of adults to recognize, report, and seek care for this type of symptom. Likewise, the fact that functional diarrhea is not formally included in the Rome IV criteria for school-aged children and adolescents may contribute to its underdiagnosis in the pediatric population, reinforcing the hypothesis that this entity is part of a continuous spectrum of DGBIs that manifests differently across stages of the life course [29].

An additional aspect that should be considered when interpreting these findings is the potential risk of symptom misclassification. Because the diagnostic framework for several of the evaluated disorders was originally developed for adults and has not been formally validated in pediatric populations, some reported symptoms could reflect overlapping gastrointestinal complaints rather than distinct clinical entities. At the same time, it is also plausible that these findings represent early phenotypic expressions of DGBIs, which may become more clearly defined later in life. From this perspective, the identification of these symptoms in children and adolescents may reflect the early stages of a continuous clinical spectrum rather than strictly misclassified conditions. Therefore, our results should be interpreted within an exploratory framework and highlight the need for future longitudinal studies to better distinguish between potential misclassification and the early manifestation of adult-defined DGBIs in pediatric populations.

One of the most consistent findings was the independent association between depressive traits and school and social absenteeism with the presence of adult DGBIs according to the Rome IV criteria in the pediatric population. From a critical perspective, these results challenge diagnostic models predominantly centered on gastrointestinal symptomatology by showing that psychoemotional and functional determinants may play an equally or even more important role than somatic factors [24,30,31,32,33,34]. Depression, in particular, may act not only as an associated factor, but also as a modulator of visceral perception thresholds and central pain processing, thereby contributing to symptom chronicity [33,34,35,36]. The interpretation of these associations should also consider the bidirectional nature of the gut–brain axis. DGBIs arise from complex and reciprocal communication between the central nervous system and the gastrointestinal tract. These interactions involve multiple biological pathways, including visceral hypersensitivity, alterations in enteric nervous system signaling, dysregulation of autonomic pathways, and neuroimmune communication within the gut–brain–microbiota axis. In this context, psychological factors such as depressive traits may influence gastrointestinal motility, visceral sensitivity, and symptom perception through central regulatory mechanisms. Conversely, persistent gastrointestinal symptoms may contribute to emotional distress, social withdrawal, and functional impairment in children and adolescents. Therefore, the relationships observed in this study should not be interpreted as unidirectional but rather as part of a dynamic and reciprocal gut–brain interaction.

School and social absenteeism emerged as a key functional marker, likely reflecting both symptom severity and the impact of these disorders on the daily life of the child or adolescent [37]. Beyond being a consequence, absenteeism may constitute a perpetuating factor of the disorder by promoting social isolation, academic stress, and emotional deterioration, thereby creating a vicious cycle that hinders clinical recovery. This aspect has been scarcely explored in pediatric studies and warrants more systematic evaluation in future research.

Although quality of life showed a significant association in the bivariate analysis, its loss of significance in the multivariate model suggests that this outcome may be mediated by psychoemotional variables, particularly depression [33,38,39]. This finding reinforces the idea that the assessment of quality of life, while fundamental, should be interpreted within a broader framework that integrates the patient’s emotional state and social functioning [39,40].

This study has important limitations that should be acknowledged. First, the temporal criterion used for symptom duration differs from that established in the original Rome IV criteria for adults. While adult Rome IV definitions generally require symptoms to be present for at least 3–6 months prior to diagnosis, the present study applied a minimum duration of 2 months, consistent with the structure of the QPGS-IV and with Rome IV pediatric diagnostic criteria. Although this approach ensured methodological consistency with the pediatric instrument used, it may have influenced the estimation of prevalence and should therefore be considered when interpreting the results. Its cross-sectional design precludes the establishment of causal relationships and does not allow for the determination of whether depressive traits precede or result from gastrointestinal symptoms. Likewise, the use of self-reported questionnaires may introduce information bias, and the application of adult criteria to a pediatric population, although conceptually justified, still lacks formal validation. These limitations, however, do not invalidate the findings; rather, they reinforce their exploratory and hypothesis-generating nature. Additionally, the adapted version of the QPGS-IV used to explore adult-defined DGBIs in this pediatric population did not undergo a formal psychometric validation process prior to its application. Although the adaptation was based on the Rome IV diagnostic framework and maintained the original questionnaire structure, the absence of standardized validation procedures (such as formal face or content validity assessment) should be considered when interpreting the results. Nevertheless, internal consistency analysis performed within the study population demonstrated a high Cronbach’s alpha values for most domains, supporting the internal reliability of the instrument in this exploratory context. Another limitation is the absence of specific socioeconomic indicators in the dataset. Although all participants were recruited from public educational institutions, which provides a relatively homogeneous educational setting, socioeconomic variability may still exist within this population and could potentially influence psychological variables such as anxiety, depression, and quality of life. These limitations, however, do not invalidate the findings; rather, they reinforce their exploratory and hypothesis-generating nature. Importantly, the diagnostic criteria applied were strictly based on Rome IV definitions, and this study represents one of the first exploratory analyses evaluating the potential presence of adult-defined DGBIs in a Latin American pediatric population.

Overall, the results of this study support a critical view of the current diagnostic approach to DGBIs in pediatrics and highlight the need to move toward more integrative and dimensional models. The systematic incorporation of psychoemotional and functional variables, along with the development of age-adapted diagnostic criteria, could improve the early identification of these disorders and optimize clinical management strategies in children and adolescents.

## 5. Conclusions

According to the results obtained through the application of the adapted QPGS-IV questionnaire, the presence of various functional conditions was identified in the studied pediatric population. These findings highlight the importance of recognizing that, although these conditions are commonly associated with adults, they may also occur in children and can have a significant impact on their quality of life and development. In addition, the main factors associated with the presence of these conditions were depressive traits and school absenteeism, suggesting a potential interaction between psychosocial and emotional aspects and the emergence of functional symptoms in the pediatric population.

These results lead to the important consideration that diagnostic criteria and classifications, such as the Rome criteria—currently focused on adults—could benefit from revision or adaptation to include these disorders in children, while recognizing their specificity at this stage of life. In addition, healthcare professionals are encouraged to consider not only pathophysiological aspects, but also psychosocial and behavioral factors that may influence the onset and persistence of these symptoms. Integrating these components into clinical assessment would allow for a more comprehensive and effective approach, promoting more personalized therapeutic strategies and improving outcomes in pediatric care. In conclusion, these findings underscore the need to broaden the diagnostic approach toward a biopsychosocial perspective, taking into account the particularities of the pediatric and adolescent population to optimize clinical management and prevention.

## Figures and Tables

**Table 1:** Comparison between DGBI diagnostic criteria in adults and screening questions for identification in children and adolescents.

Disorder	Definition According to the Rome Criteria in Adults	Questions and Answers Added to the Pediatric QPGS-IV, Present During the Past Month…
Functional dysphagia	1. Persistent or recurrent sensation of difficulty in the passage of solid and/or liquid foods 2. Absence of evidence of structural, mucosal, or motor abnormalities explaining the symptom 3. The symptoms are not explained by GERD * or major esophageal motility disorders 4. Symptoms must be present during the last 3 months, with onset at least 6 months prior to diagnosis	**Choking sensation** Does food or drink get stuck in your chest after swallowing, or does it pass slowly through your chest? How many days? = 4 or more days And For how long? = 2 or more months
Functional diarrhea	1. Predominantly loose or liquid stools without predominant stomachache 2. Increased frequency of bowel movements 3. Absence of criteria for irritable bowel syndrome with diarrhea 4. Symptoms must be present during the last 3 months, with onset at least 6 months prior to diagnosis	**Diarrhea** How often does he/she poop? 3 to 6 times per week, or Once a day, or Two to three times a day, or More than 3 times a day And How is poop according to the Bristol Stool Scale? = Type 6 or 7 And Does it hurt when you poop? = No And What is your main complaint? Stomachache, or Loose/mucous stool or stool with undigested food, or Frequent bowel movements, or Bloated stomach And Does he/she gain weight normally? = Yes
Functional chest pain	1. Recurrent retrosternal pain or discomfort 2. No evidence of heart disease as the cause of the pain 3. Absence of structural, inflammatory, or motor esophageal disease that explains the symptoms 4. The symptoms are not explained by GERD * 5. Symptoms must be present during the last 3 months, with onset at least 6 months prior to diagnosis	**Chest pain or discomfort** How many days? = 4 or more days And For how long? = 2 months or more, And How often does he/she experience a burning sensation? = Never And How frequently is it associated with food choking when swallowing? Occasionally, or Sometimes, or Most of the time, or Always
Functional biliary pain	1. Recurrent episodes of pain located in the epigastrium and/or right upper quadrant 2. The pain reaches a steady intensity and lasts ≥30 min 3. The pain interferes with daily activities or leads to medical consultation 4. It is not relieved by bowel movements, postural changes, or antacids 5. Absence of gallstones or other structural pathology 6. Symptoms must be present during the last 3 months, with onset at least 6 months prior to diagnosis	**Pain or discomfort in the right upper abdomen quadrant, even if of short duration:** How many days? = 4 or more days And For how long? = 2 months or more, And As the hours go by, does he/she improve? = No And As the hours go by, does he/she get worse? = Yes And Does the pain occur at the same time as pooping? = Never And Was the poop softer, or more liquid, than usual? = Never And Was the stool harder or in pieces, than usual? = Never And Does he/she poop more times, than usual? = Never And Does he/she poop less times, than usual? = Never And Does it improve with medications? = Never And Suspension of activities or visit to a doctor or emergency room? = Yes And For how long were the activities suspended? Between half an hour and 1 h, or 1–2 h, or 3–4 h, or Most of the day, or All day And Does it improve by changing position from lying down to sitting? = No And/or Does it improve by changing position from sitting to standing? = No And How long does it take before it repeats? Several hours, or Several days, or Several weeks, or Several months
Functional heartburn	1. Recurrent retrosternal burning sensation 2. Absence of evidence of GERD * 3. Absence of esophageal motility disorders or structural disease 4. Symptoms do not respond adequately to antisecretory therapy 5. Symptoms must be present during the last 3 months, with onset at least 6 months prior to diagnosis	**Heartburn (burning, stinging, discomfort) in the chest:** How many days? = 4 or more days And For how long? = 2 months or more, And Medications for reflux? = Yes And Does it improve with medication? Never, or Occasionally, or Sometimes, or Most of the time, or
Proctalgia fugax	1. Recurrent episodes of intense, short-duration rectal pain (seconds to minutes) 2. Absence of anorectal pain between episodes 3. Absence of anorectal structural or inflammatory cause 4. It is not related with bowel movements 5. Symptoms must be present during the last 3 months, with onset at least 6 months prior to diagnosis	**Pain, discomfort, or pressure in the rectum without having a bowel movement:** How many days? = 4 or more days And For how long? = 2 months or more, And How long does it last? = More than a minute, but less than 30 min And How long does it take before it repeats? Several hours, or Several days, or Several weeks, or Several months

Reprinted/adapted with permission from Ref. [13]. Drossman, D.A., 2016. * GERD = Gastroesophageal Reflux Disease.

**Table 2:** DGBIs in adults according to the Rome IV criteria presented in toddlers, school-aged children and adolescents from three Colombian public educational institutions. N = 704.

DGBIs in Adults According to the Rome IV Criteria Presented in Children		95%CI
No	663 (94.2)	0.92–0.95
Yes	41 (5.8)	0.04–0.07
Proctalgia fugax	20 (2.8)	0.01–0.04
Functional heartburn	7 (1.0)	0.00–0.02
Functional diarrhea	6 (0.9)	0.00–0.01
Functional dysphagia	4 (0.6)	0.00–0.01
Functional chest pain	3 (0.4)	0.00–0.01
Functional biliary pain	1 (0.1)	0.00–0.00

**Table 3:** Characteristics of children with adult DGBIs according to the Rome IV criteria presented at three Colombian public educational institutions. N = 789.

	All (n = 789)	Functional Chest Pain (n = 3)	Functional Biliary Pain (n = 1)	Proctalgia Fugax (n = 20)	Functional Heartburn (n = 7)	Functional Dysphagia (n = 4)	Functional Diarrhea (n = 6)
**Sociodemographic variables**							
**Age (years)**							
X ± SD	13.7 ± 2.8	14.2 ± 2.6	16.1	13.8 ± 3.7	13.7 ± 2.5	15.3 ± 2.3	14.4 ± 1.6
Range	4–18	11–16	n/a	4–18	10–17	13–18	12–16
**Age groups**							
Toddlers	9 (1.1)	0 (0.0)	0 (0.0)	1 (5.0)	0 (0.0)	0 (0.0)	0 (0.0)
School-aged children	214 (27.1)	1 (33.3)	0 (0.0)	4 (20.0)	3 (42.9)	0 (0.0)	0 (0.0)
Adolescents	566 (71.8)	2 (66.7)	1 (100.0)	15 (75.0)	4 (57.1)	4 (100.0)	6 (100.0)
**Sex**							
Female	372 (47.2)	2 (66.7)	0 (0.0)	12 (60.0)	2 (28.6)	4 (100.0)	3 (50.0)
Male	417 (52.8)	1 (33.3)	1 (100.0)	8 (40.0)	5 (71.4)	0 (0.0)	3 (50.0)
**City**							
Cucuta	358 (45.4)	1 (33.3)	0 (0.0)	9 (45.0)	5 (71.4)	1 (25.0)	0 (0.0)
Corozal	270 (34.2)	1 (33.3)	0 (0.0)	9 (45.0)	0 (0.0)	0 (0.0)	6 (100.0)
Maicao	161 (20.4)	1 (33.3)	1 (100.0)	2 (10.0)	2 (28.6)	3 (75.0)	0 (0.0)
**Race**	**(n = 716)**	**(n = 3)**	**(n = 1)**	**(n = 20)**	**(n = 7)**	**(n = 4)**	**(n = 6)**
Mixed race	364 (50.8)	3 (100.0)	1 (100.0)	4 (20.0)	1 (14.3)	2 (50.0)	1 (16.7)
White	221 (30.9)	0 (0.0)	0 (0.0)	9 (45.0)	6 (85.7)	1 (25.0)	2 (33.3)
Indigenous	102 (14.3)	0 (0.0)	0 (0.0)	7 (35.0)	0 (0.0)	1 (25.0)	3 (50.0)
Afro-descendant	29 (4.0)	0 (0.0)	0 (0.0)	0 (0.0)	0 (0.0)	0 (0.0)	0 (0.0)
**Clinical Variables**							
**COVID-19**	**(n = 704)**	**(n = 3)**	**(n = 1)**	**(n = 20)**	**(n = 7)**	**(n = 4)**	**(n = 6)**
**History of COVID-19**							
No	658 (93.5)	1 (33.3)	1 (100.0)	19 (95.0)	6 (85.7)	4 (100.0)	6 (100.0)
Yes	46 (6.5)	2 (66.7)	0 (0.0)	1 (5.0)	1 (14.3)	0 (0.0)	0 (0.0)
**COVID-19 vaccination**	**(n = 660)**	**(n = 3)**	**(n = 1)**	**(n = 19)**	**(n = 7)**	**(n = 4)**	**(n = 6)**
No	356 (53.9)	0 (0.0)	0 (0.0)	15 (78.9)	4 (57.1)	1 (25.0)	5 (83.3)
Yes	304 (46.1)	3 (100.0)	1 (100.0)	4 (21.1)	3 (42.9)	3 (75.0)	1 (16.7)
**School/social absenteeism**							
**School absenteeism**	**(n = 704)**	**(n = 3)**	**(n = 1)**	**(n = 20)**	**(n = 7)**	**(n = 4)**	**(n = 6)**
No	527 (74.9)	1 (33.3)	0 (0.0)	8 (40.0)	4 (57.1)	1 (25.0)	5 (83.3)
Si	177 (25.1)	2 (66.7)	1 (100.0)	12 (60.0)	3 (42.9)	3 (75.0)	1 (16.7)
**Reason for school absenteeism**							
Abdominal pain	18 (10.2)	1 (50.0)	0 (0.0)	5 (41.7)	1 (33.3)	1 (33.3)	1 (100.0)
Headache	16 (9.0)	1 (50.0)	0 (0.0)	2 (16.7)	0 (0.0)	1 (33.3)	0 (0.0)
Nausea	11 (6.2)	0 (0.0)	0 (0.0)	3 (25.0)	1 (33.3)	0 (0.0)	0 (0.0)
Colic	11 (6.2)	0 (0.0)	1 (100.0)	1 (8.3)	1 (33.3)	1 (33.3)	0 (0.0)
Vomiting	9 (5.1)	0 (0.0)	0 (0.0)	1 (8.3)	0 (0.0)	0 (0.0)	0 (0.0)
Other	112 (63.3)	0 (0.0)	0 (0.0)	0 (0.0)	0 (0.0)	0 (0.0)	0 (0.0)
**Social absenteeism**	**(n = 704)**	**(n = 3)**	**(n = 1)**	**(n = 20)**	**(n = 7)**	**(n = 4)**	**(n = 6)**
No	552 (78.4)	1 (33.3)	0 (0.0)	8 (40.0)	3 (42.9)	4 (100.0)	5 (83.3)
Yes	152 (21.6)	2 (66.7)	1 (100.0)	12 (60.0)	4 (57.1)	0 (0.0)	1 (16.7)
**Social activity**							
Go out with friends	35 (23.0)	0 (0.0)	0 (0.0)	4 (20.0)	1 (25.0)	0 (0.0)	0 (0.0)
Go out with family	21 (13.8)	0 (0.0)	0 (0.0)	4 (20.0)	1 (25.0)	0 (0.0)	1 (50.0)
Play	32 (21.0)	0 (0.0)	1 (100.0)	2 (10.0)	2 (50.0)	0 (0.0)	0 (0.0)
Play a sport	37 (24.3)	1 (50.0)	0 (0.0)	2 (10.0)	0 (0.0)	0 (0.0)	0 (0.0)
Other	27 (17.8)	1 (50.0)	0 (0.0)	0 (0.0)	0 (0.0)	0 (0.0)	0 (0.0)
**Quality of life**							
**Quality of life according to PedsQL 4.0**	**(n = 580)**	**(n = 3)**	**(n = 1)**	**(n = 17)**	**(n = 4)**	**(n = 4)**	**(n = 6)**
Normal	414 (71.4)	0 (0.0)	n/a	11 (64.7)	2 (50.0)	1 (25.0)	4 (66.7)
Altered	166 (28.6)	3 (100.0)		6 (35.3)	2 (50.0)	3 (75.0)	2 (33.3)
**Anxiety traits**	**(n = 451)**	**(n = 2)**		**(n = 13)**	**(n = 3)**	**(n = 3)**	**(n = 6)**
None to mild	335 (74.3)	1 (50.0)	n/a	6 (46.2)	1 (33.3)	0 (0.0)	2 (33.3)
Mild	80 (17.7)	1 (50.0)		3 (23.1)	1 (33.3)	1 (33.3)	3 (50.0)
Moderate	31 (6.9)	0 (0.0)		4 (30.8)	1 (33.3)	2 (66.7)	1 (16.7)
Severe	5 (1.1)	0 (0.0)		0 (0.0)	0 (0.0)	0 (0.0)	0 (0.0)
**Depressive traits**	**(n = 462)**	**(n = 2)**		**(n = 16)**	**(n = 3)**	**(n = 3)**	**(n = 6)**
None to mild	314 (68.0)	1 (50.0)	n/a	6 (37.5)	2 (66.7)	0 (0.0)	2 (33.3)
Mild	98 (21.2)	0 (0.0)		7 (43.8)	0 (0.0)	1 (33.3)	4 (66.7)
Moderate	46 (10.0)	0 (0.0)		3 (18.8)	1 (33.3)	2 (66.7)	0 (0.0)
Severe	4 (0.9)	1 (50.0)		0 (0.0)	0 (0.0)	0 (0.0)	0 (0.0)

SD = standard deviation, PedsQL 4.0 = Pediatric Quality of Life Inventory™ 4.0.

**Table 4:** Possible associations of DGBIs in adults present in toddlers, school-aged children and adolescents from three Colombian public educational institutions. N = 41.

	DGBIs in Adults				
	No	Yes	OR	95%CI	*p*
	n = 663	n = 41			
**Race**					
**Indigenous**					
No	574 (86.6)	30 (73.2)	1.00		0.0170
Yes	89 (13.4)	11 (26.8)	2.36	1.02–5.06	
**School absenteeism**					
No	508 (76.6)	19 (46.3)	1.00		*p* < 0.001
Yes	155 (23.4)	22 (53.7)	3.79	1.90–7.60	
**Social absenteeism**					
No	535 (80.7)	17 (41.5)	1.00		*p* < 0.001
Yes	128 (19.3)	24 (58.5)	5.90	2.93–12.03	
**Depressive traits**	**(n = 419)**	**(n = 30)**			
No	292 (69.7)	11 (36.7)	1.00		0.0002
Yes	127 (30.3)	19 (63.3)	3.97	1.73–9.49	
**Quality of life**	**(n = 534)**	**(n = 34)**			
Normal	388 (72.7)	18 (52.9)	1.00		0.0135
Altered	146 (27.3)	16 (47.1)	2.36	1.09–5.04	

DGBIs = disorders of gut–brain interaction, OR = odds ratios, 95%CI = 95% confidence interval.

**Table 5:** Logistic regression analysis.

	OR	95%CI	*p*
Depressive traits	4.08	1.82–9.12	0.001
School absenteeism	2.51	1.06–5.98	0.036
Social absenteeism	4.04	1.70–9.62	0.002

OR = odds ratios, 95%CI = 95% confidence interval.

## Data Availability

The data presented in this study are available on request from the corresponding author. The data are not publicly available due to privacy and ethical restrictions.

## Abbreviations

The following abbreviations are used in this manuscript:

DGBIs	Disorders of the gut–brain interaction
QPGS-IV	Questionnaire for Pediatric Gastrointestinal Symptoms Rome IV
GERD	Gastroesophageal reflux disease
PedsQL	Pediatric Quality of Life Inventory™ Questionnaire
PROMIS	Patient-Reported Outcomes Measurement Information System
95%CIs	95% confidence intervals
ORs	Odds ratios
SD	Standard deviation
